# A novel predictive model based on inflammatory markers to assess the prognosis of patients with HBV-related acute-on-chronic liver failure: a retrospective cohort study

**DOI:** 10.1186/s12876-020-01437-2

**Published:** 2020-09-16

**Authors:** Li Qiang, Jiao Qin, Changfeng Sun, Yunjian Sheng, Wen Chen, Bangdong Qiu, Xin Chen, Yuanfang Chen, Fei Liu, Gang Wu

**Affiliations:** 1 Department of Infectious disease, The Affiliated Hospital of Southwest Medical University, Luzhou City, 646000 Sichuan Province China; 2 Department of Infectious disease, Public Health Clinical Center of Chengdu, Chengdu City, 610000 Sichuan Province China; 3 Department of Infectious disease, The Second People’s Hospital of Yibin, Yibin City, 644000 Sichuan Province China; 4 Department of Infectious disease, The First People’s Hospital of Neijiang, Neijiang City, 641000 Sichuan Province China

**Keywords:** Liver failure, Hepatitis B virus, Prediction model, Inflammatory markers, Red blood cell distribution width, Neutrophil/lymphocyte ratio

## Abstract

**Background:**

Systemic inflammatory response is closely related to the development and prognosis of liver failure. This study aimed to establish a new model combing the inflammatory markers including neutrophil/lymphocyte ratio (NLR) and red blood cell distribution width (RDW) with several hematological testing indicators to assess the prognosis of patients with hepatitis B virus-related acute-on-chronic liver failure (HBV-ACLF).

**Methods:**

A derivation cohort with 421 patients and a validation cohort with 156 patients were recruited from three hospitals. Retrospectively collecting their clinical data and laboratory testing indicators. Medcalc-15.10 software was employed for data analyses.

**Results:**

Multivariate analysis indicated that RDW, NLR, INR, TBIL and Cr were risk factors for 90-day mortality in patients with HBV-ACLF. The risk assessment model is COX_RNTIC_ = 0.053 × **R**DW + 0.027 × **N**LR + 0.003 × **T**BIL+ 0.317 × **I**NR + 0.003 × **C**r (RNTIC) with a cut-off value of 3.08 (sensitivity: 77.89%, specificity: 86.04%). The area under the receiver operating characteristics curve (AUC) of the RNTIC was 0.873 [95% CI(0.837–0.903)], better than the predictive value of MELD score [0.732, 95% CI(0.687–0.774)], MELD-Na [0.714, 95% CI(0.668–0.757)], CTP[0.703, 95% CI(0.657–0.747)]. In the validation cohort, RNTIC also performed a better prediction value than MELD score, MELD-Na and CTP with the AUC of [0.845, 95% CI(0.778–0.898)], [0.768, 95% CI (0.694–0.832)], [0.759, 95% CI(0.684–0.824)] and [0.718, 95% CI(0.641–0.787)] respectively.

**Conclusions:**

The inflammatory markers RDW and NLR could be used as independent predictors of 90-day mortality in patients with HBV-ACLF. Compared with MELD score, MELD-Na and CTP, RNTIC had a more powerful predictive value for prognosis of patients with HBV-ACLF.

## Background

Acute-on-chronic liver failure (ACLF), a series of clinical syndrome resulted from culmination of chronic liver disease leading to single or multiple organ failures, has been shown to carry poor prognosis with a short-term mortality of > 50% [1]. At present, conservative medical treatment usually has been adopted, due to the artificial liver support system is poorly effective for end-stage liver failure, whereas stem cell therapy is still in development and faced with ethical issues [2]. Moreover, most patients with end-stage liver failure are suffering with multi-system organ failures resulting in many limitations in liver transplantation [3]. Therefore, reliable, user-friendly, inexpensive and reproducible predictors of survival are important to evaluate the risk of death early and choose treatment appropriately in those patients.

Currently, amounts of predictive scoring systems are available for assessing the prognosis in patients with ACLF, including chronic liver failure sequential organ failure assessment (CLIF-SOFA) score, Child-Turcotte Pugh (CTP) score, model for end-stage liver disease (MELD) score, MELD-sodium (MELD-Na) score [4]. The Model for End-Stage Liver Disease (MELD) score has the advantage of objective parameters which is often used for the prognosis of the patients with ACLF. In China, most cases of ACLF are caused by hepatitis B virus (HBV) infection, but those scores were established in European and American countries, where the alcohol is the most leading cause of the ACLF. Since these kinds of scoring systems might have certain limitations for HBV-related acute-on-chronic liver failure (HBV-ACLF), this study intended to establish a new model applicable to patients in China.

Nowadays, increasing evidences showed that systemic inflammatory response played a pivotal role in the development of liver failure and cirrhosis [5, 6]. A generalized activation of the inflammatory cytokines not only resulting to an accentuation of systemic circulatory dysfunction and organ hypo-perfusion, but also directly doing harm to organ function [7]. Inflammatory cytokines could affect the survival of erythrocytes, suppress maturation, lead larger and newer reticulocytes to enter circulation and increase the RDW [8]. The elevated granulocyte colony stimulating factorand granulocyte-macrophage colony stimulating factor, key regulatory cytokines that target committed progenitors promote differentiation and activation of monocytes and neutrophils [9]. Interleukin-6 (IL-6), an increased pro-inflammation cytokine in HBV-ACLF patients, also has ability to lead amounts of young platelets in the bone marrow to be released to the bloodstream [10] thus making the mean platelet volume (MPV) elevated [11]. The occurrence of ACLF generally represents a complicated state of host immune dysregulation. Excessive immune activation could lead to a decrease in lymphocyte numbers caused by activation induced cell death and impaired lymphopoiesis [12]. Based on the large amounts of investigations on systemic inflammation, routine hematology parameters, neutrophil/lymphocyte ratio (NLR), monocyte/lymphocyte ratio (MLR), platelet/lymphocyte ratio (PLR), red cell distribution width (RDW), RDW/platelet ratio (RPR), gamma-glutamyl transpeptidase/platelet ratio (GPR), mean platelet volume (MPV), RDW/lymphocyte ratio (RLR) and prognostic nutritional index (PNI) and MPV/platelet ratio (MPR), are being considered as the inflammatory markers which could predict outcomes of various diseases [13–15]. Thus, this study aimed to identify inflammatory markers and hematological indicators associated with a short-term negative prognosis and establish a new multi-factor combined prognostic model for patients with HBV-ACLF.

## Methods

### Patient selection

ACLF was defined as the acute deterioration of liver function manifested as jaundice [total bilirubin (TBIL) ≥5 mg/dL or ≥ 85 μmol/L and coagulopathy with international normalized ratio of prothrombin time (INR) ≥1.5 or prothrombin activity (PTA) ≤40%, complicated with ascites and/or hepatic encephalopathy noted within 4 weeks in a patient diagnosed with HBV related chronic liver disease/cirrhosis [1]. All patients also meet the Chinese guidelines for diagnosis and treatment of liver failure [16]. The cirrhosis was diagnosed histologically proven or clearly considered on the basis of biological, clinical, and radiological features. Patients with cardiac diseases, endocrinological disorders, hematological disease and other types of cancer were excluded. Co-infection with human immunodeficiency virus, hepatitis A, C, D, and E viruses or other hepatitis viruses, autoimmune diseases, alcoholic liver disease, acute liver failure, drug-induced liver injury, coexistent hepatocellular carcinoma, and any other serious medical illness or patients who had received any immunotherapy, liver transplantation or artificial liver support were also excluded. The enrolled patients all hospitalized in the ward of infectious department, there were none patients on dialysis under mechanical ventilation.

421 patients admitted to the Affiliated Hospital of Southwest Medical University were consecutively recruited as a derivation cohort to establish the new prognostic model between January 1, 2014 to February 28, 2019. 56 patients in the First People’s Hospital of Neijiang and 100 patients in the Second People’s Hospital of Yibin from January 1, 2017 to February 28, 2019 were enrolled as a validation cohort. Retrospectively collecting their clinical data and baseline laboratory testing indicators and tracking patients’ survivals by telephone and clinical follow-up until May 01, 2019 to ensure that the last case (enrolled before February 01, 2019) was also followed up the 90-day survival.

All patients admitted were given a standard medical treatment including nutritional support, antiviral therapy, intravenous infusion albumin and plasma, treatment of complications.

### Clinical data collection

Clinical data included age, gender, telephone number, temperature, oxygen saturation, blood pressure, hepatic encephalopathy (HE), liver cirrhosis and type of infection were retrospectively abstracted from the medical record. The HE was diagnosed according to the West-Haven criteria [17]. Ascites and HE were the situation when patients were at admission. Bacterial infections were collected during the entire hospital stay. The diagnosis of bacterial infection was based on infection-positive cultures of blood, ascites, urine or sputum, and/or clinical symptoms suggestive of infections.

### Laboratory analysis

Demographic and clinical characteristics of the included patients were recorded. Blood samples were collected from an antecubital vein after overnight fasting on the first day of admission, and detected the complete blood counts and biochemical tests by Mind 6800 automated blood analyzer and Mindray BS200 biochemical analyzer, respectively. Coagulation indicators were assessed using a CS-5100 automated coagulation analyzer. The HBV-DNA levels in serum were quantified by ABI 7500FAST (fluorescence quantitative PCR). The above results were from the test departments of three hospitals with a high reliability. The MELD score was calculated using the Kamath formula: R = 9.6 × ln(Cr mg/dl) + 3.8 × ln(TBIL mg/ dl) + 11.2 × ln(INR) + 6.4 [18]. PNI = albumin (g/L) + 5 × lymphocyte count (10^9^/L). MELD-Na = MELD+ 1.59 × [135-Na (mmol/l)] in accordance with Biggins et al. [19]. CTP including HE, prothrombin time (PT), ascites, TBIL, and serum albumin was assessed according to the standard criteria [20].

### Statistical analysis

Normally distributed variables were expressed as means± standard deviation (SD), and non-normally distributed variables were expressed as a median and interquartile range (IQR). Count and percentages were used to describe categorical variables. Two independent groups were compared using the t test for continuous normally-distributed variables and the Mann–Whitney U test for non-normally distributed variables. For categorical variables, comparisons between groups used the Chi-squared test or the Fisher test as appropriate. The Kaplan–Meier method was used to calculate the 90-day survival probability curves. The BOX-Tidwell method was used to test the wireless relationship between the independent and dependent variable. The tolerance and variance expansion factor were used to test the multicollinearity between the independent variables. Cox regression models were used for univariate and multivariate analysis of outcome predictors. Cut-off values were determined via the receiver operating characteristic (ROC) analysis. All calculations were performed by MedCalc software (version15.10). *P* values< 0.05 based on a two-tailed test were considered with statistically significance.

## Results

### Basic characteristics of patients with HBV-ACLF in derivation cohort

**Fig. 1:**
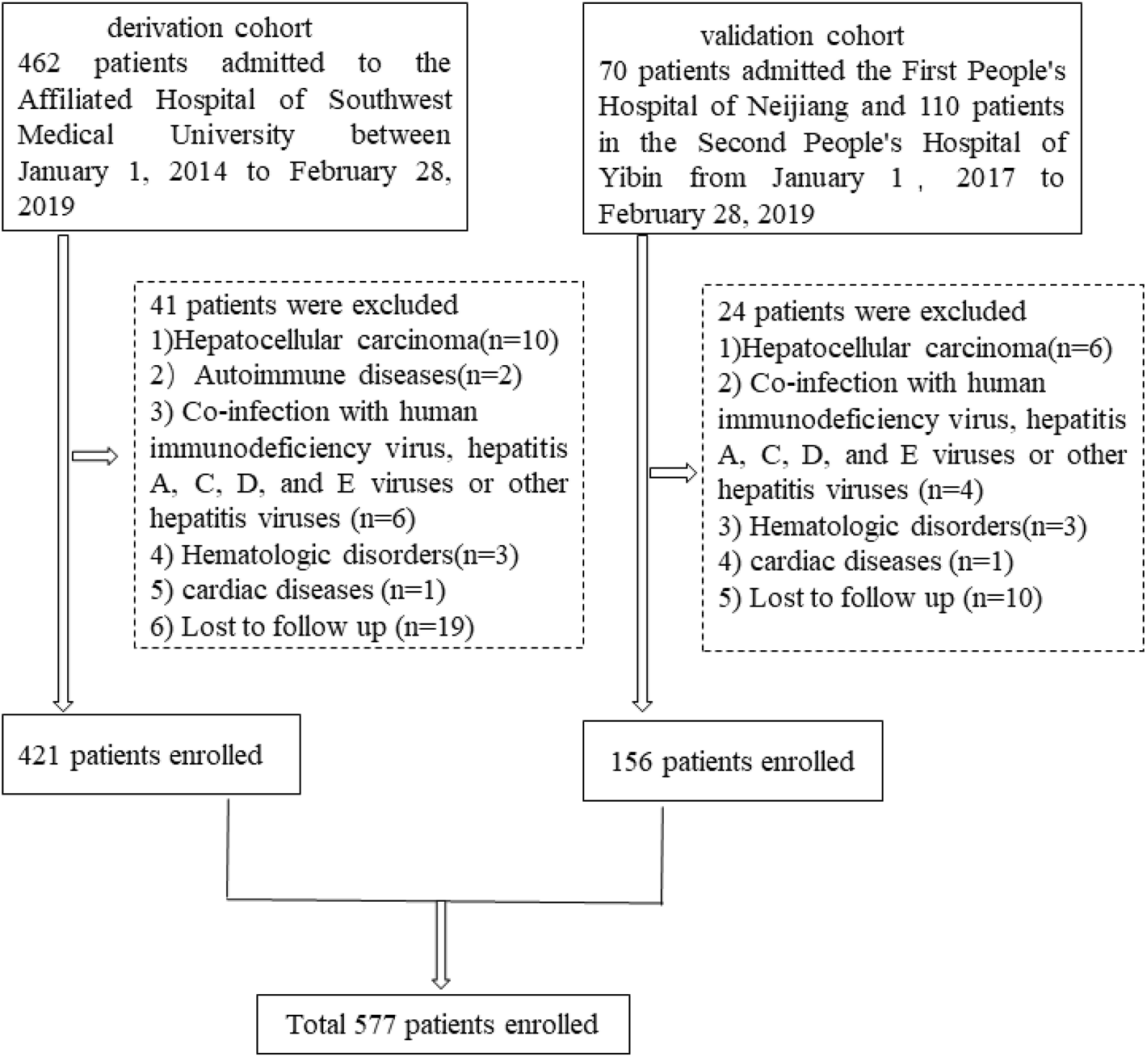
A flow diagram of study participants

A total of 642 HBV-ACLF patients were collected from 3 centers. According to the inclusion and exclusion criteria, 65 patients were excluded (Fig. 1). Finally, 577 patients with HBV-ACLF were enrolled in the study. Among them, 421 patients from the affiliated hospital of southwest medical university were derivation cohort, and basic characteristics of patients were listed in Table 1.Besides, in the derivation cohort, 307 (72.92%) patients complicated with bacterial infection, in whom 245(58.19%) had a single infection site, 41(9.73%) had 2 infection sites, and 21 (4.99%) had ≥3 infection sites on admission. The most frequent infection was pneumonia (*n* = 188, 44.66%), followed by SBP (*n* = 167, 39.67%) and intestinal infection (*n* = 36, 8.55%).

**Table 1:** Comparisons of characteristics between survivors and non-survivors in patients with HBV-ACLF

Variables	Total patients (*n* = 421)	Survivors (*n* = 222)	Non-survivors (*n* = 199)	*P* value
Age (years)	47.93 ± 11.40	48.10 ± 11.20	47.75 ± 11.64	0.735
Gender (M/F)	365/56	195/27	170/29	0.468
Cirrhosis (%)	299(71.02%)	159(71.62)	140(70.35)	0.744
Bacterial infections	307(72.92%)	145(65.32)	162(81.41)	< 0.001
HBV-DNA (10^7^IU/mL)	1.53 ± 4.85	1.89 ± 5.16	1.17 ± 4.53	0.139
HbeAg(+) n (%)	116(27.55)	67(30.18)	49(24.62)	0.203
ALT(U/L)	228(63.20,847.60)	258.75(52.65,1000.33)	198.20(74.80,755.10)	0.532
AST(U/L)	223.40(103.95,641.90)	254.10(94.63,254.10)	206.10(110.10,511.20)	0.477
TBIL(μmol/L)	323.39 ± 165.69	236.35 ± 118.55	420.49 ± 156.77	< 0.001
ALB(g/L)	29.09 ± 5.57	29.33 ± 5.81	28.82 ± 5.30	0.348
γ-GGT(U/L)	85.40(48.5143.00)	79.35(48.45,157,13)	86.30(48.50,126.80)	0.719
Cr (μmol/L)	69.30(57.25,87.90)	68.45(57.90,79.78)	70.1(55.6106.8)	0.002
Cyst-c(mg/L)	1.21(0.92,1.756)	1.11(0.89,1.55)	1.38(0.93,1.38)	0.001
K^+^(mmol/L)	3.97 ± 0.65	3.99 ± 0.60	3.95 ± 0.70	0.510
Na^+^(mmol/L)	135.46 ± 5.71	135.79 ± 5.70	134.86 ± 5.66	0.042
PT(s)	24.90(21.35,29.30)	23.70(21.38,27.88)	26.40(21.3,31.7)	0.271
INR	2.25(1.86,2.76)	2.12(1.86,2.62)	2.40(1.85,3.16)	< 0.001
PTA (%)	34.11 ± 10.60	35.79 ± 9.49	32.23 ± 11.45	0.001
WBC (10^9^/L)	6.43(4.72,9.13)	5.83(424,7.86)	7.56(5.45,10.71)	< 0.001
Neutrophils (10^9^/L)	4.65(3.11,7.21)	3.92(2.88,5.80)	5.98(3.82,8.52)	< 0.001
Lymphocytes(10^9^/L)	0.96(0.66,1.33)	1.03(0.71,1.41)	0.89(0.61,1.28)	0.030
Monocytes(10^9^/L)	0.61(0.40,0.90)	0.54(0.37,0.77)	0.70(0.45,1.04)	< 0.001
RDW (%)	15.90(14.35,18.45)	15.00(14.0,17.03)	16.82(15.00,20.70)	< 0.001
PLT (10^9^/L)	86.00 (58.0,126.0)	82.00(53.0,125.0)	89.00(64.0,89.0)	0.155
MPV(FL)	11.75 ± 1.53	11.82 ± 1.48	11.67 ± 1.58	0.312
NLR	4.84(3.19,8.09)	4.00(2.57,6.10)	6.31(4.19,10.25)	< 0.001
GPR	0.97(0.59,1.69)	0.98(0.62,1.69)	0.95(0.51,1.70)	0.253
MLR	0.64(0.42,0.94)	0.53(0.38,0.74)	0.77(0.52,1.10)	< 0.001
RPR	0.19(0.12,0.30)	0.18(0.12,0.32)	0.19(0.14,0.30)	0.490
MPR	0.14(0.09,0.20)	0.15(0.10,0.22)	0.13(0.09,0.19)	0.103
PNI	34.10(30.20,38.33)	34.98(30.33,39.14)	33.65(30.10,37.30)	0.060
PLR	91.03(62.66,127.75)	85.20(57.45,118.76)	102.15(70.37,144.12)	0.001
RLR	16.70(11.66,25.96)	15.68(10.66,22.65)	20.57(13.25,30.00)	< 0.001
PCT (μg/L)	0.88(0.48,3.31)	0.79(0.44,2.88)	0.99(0.54,3.52)	0.064
MELD SCORE	25.05 ± 6.75	22.47 ± 4.67	27.94 ± 7.52	< 0.001
MELD-Na	24.43 ± 12.48	20.36 ± 11.09	28.98 ± 12.41	< 0.001
CTP, n (%)				< 0.001
5–6	1(0.24)	1(0.45)	0(0)	
7–9	69(16.39)	56(25.21)	13(6.53)	
≥ 10	351(83.37)	165(74.32)	186(93.46)	
HE, n (%)				0.005
Stage 0	332(78.86)	184(82.90)	148(74.37)	
Stage 1	14(3.33)	9(4.10)	5(2.51)	
Stage 2	23(5.46)	12(5.4)	11(5.53)	
Stage 3	28(6.65)	13(5.9)	15(7.54)	
Stage 4	24(5.70)	4(1.8)	20(10.05)	
Antiviral therapy, n (%)				0.841
ETV	339(80.52)	175(78.83)	164(82.41)	
TDF	56(13.30)	32(14.41)	24(12.06)	
LAM	10(2.38)	5(2.25)	5(2.51)	
ADV	12(3.09)	7(3.15)	5(2.51)	
ADV + LAM	4(0.95)	3(1.35)	1(0.50)	

*ALT* alanine aminotransferase, *AST* aspartate aminotransferase, *TBIL* total bilirubin, *ALB* albumin, *γ-GGT* gamma-glutamyl transpeptidase, *Cr* creatinine, *Cyst-c* cystatin c, *Serum k*^*+*^ serum potassium, *Serum Na*^*+*^ serum sodium, *PT* prothrombin time, *INR* international normalized ratio, *PTA* prothrombin activity, *WBC* white blood cell count, *RBC* red blood cells, *HGB* hemoglobin, *PLT* platelet, *RDW* red blood cell distribution width, *NLR* neutrophil/lymphocyte ratio, *MLR* monocyte/lymphocyte ratio, *PLR* platelet/lymphocyte ratio, *RPR* RDW/platelet ratio, *GPR* gamma-glutamyl transpeptidase/platelet ratio, *MPV* mean platelet volume, *RLR* RDW/lymphocyte ratio, *PNI* prognostic nutritional index, *MPR* MPV/platelet ratio, *PCT* procalcitonin, *MELD SCORE* model for end-stage liver disease score, *CTP* child-Turcotte Pugh score, *MELD-Na* MELD-sodium score, *HE* hepatic encephalopathy, *ETV* entecavir, *TDF* tenofovirdisoproxil, *LAM* lamivudine, *ADV* adefovir dipivoxil

### Comparison of inflammatory markers and routine hematological parameters between survivors and non-survivors

In order to identify indicators with statistical differences, inflammatory markers and routine hematological parameters in the survivors and non-survivors were analyzed. For the inflammatory markers, compared with the survivors, the level of NLR, MLR, PLR, RLR and RDW increased (*P* ≤ 0.001) (Table 1); For the routine hematological parameters, compared with the survivors, the serum Na, PTA and lymphocytes were lower, while WBC, neutrophils, monocytes, TBIL, Cr, cyst-c, PT, INR, MELD scores, MELD-Na and CTP were higher (*P*<0.05). Moreover, the incidence of hepatic encephalopathy was elevated in the non-survivors (*P* < 0.05) (Table 1).

### Univariate and multivariate cox regression analysis of survival and death in HBV-ACLF patients

**Fig. 2:**
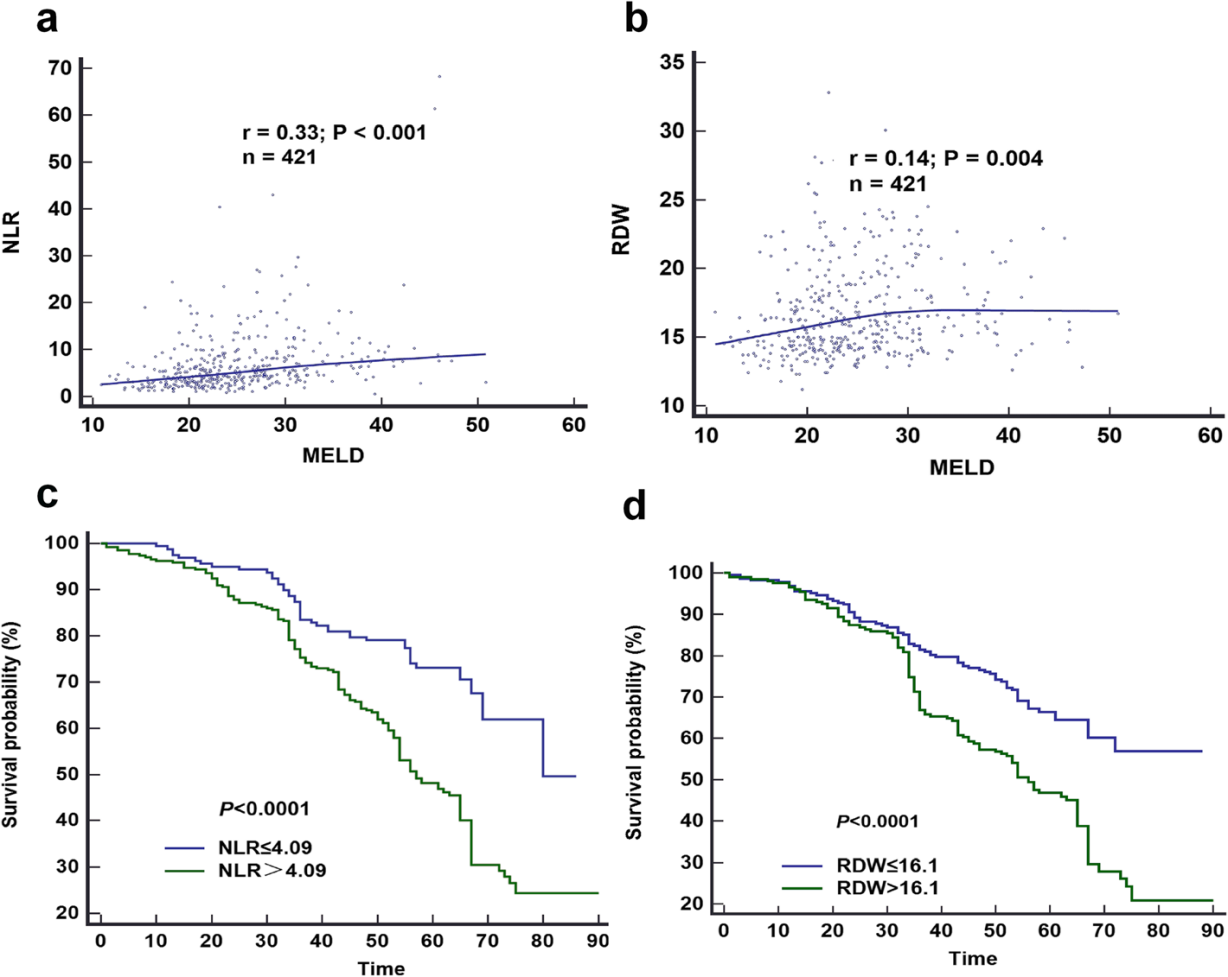
NLR(**a**) and RDW(**b**) levels correlated with MELD score in patients with HBV-ACLF, respectively; (**c**) The survival curves of groups of NLR>4.09 and NLR ≤ 4.09 by Kaplan-Meier survival analysis; (**d**) The survival curves of groups of RDW>16.1 and RDW ≤ 16.1 by Kaplan-Meier survival analysis

Univariate regression analysis was performed on statistic significant indicators in Table 1, and multivariate cox regression analysis was performed on the indicators with significant difference in univariate analysis (*P* < 0.05), including TBIL, Cr, Cyst-c, INR, PTA, WBC, neutrophils, RDW, NLR, RLR, PLR, MLR. The multivariate cox regression results indicated that RDW, NLR, TBIL, INR, Cr were risk factors for 90-day death in HBV-ACLF patients (*P* < 0.05). In addition, RDW and NLR were significantly positively correlated with MELD scores (*P* < 0.05), suggesting that high RDW, NLR might be closely associated with the prognosis of the patients with HBV-ACLF (Fig. 2a and b). We further identified the patients with HBV-ACLF based on the cut-off values of NLR and RDW to graph the Kaplan-Meier survival curves. The results showed that the patients with NLR>4.09 and RDW>16.10 had a more worse prognosis (Fig. 2c and d).

### Establishing a new prognostic model combining inflammatory markers with hematological parameters in patients with HBV-ACLF by cox regression

The two inflammatory markers RDW, NLR and other three hematological parameters TBIL, INR, Cr had been found to be related to the prognosis of patients with HBV-ACLF in forward analysis. Based on the regression coefficient (Beta coefficient) as the weight of the risk factor (Table 2), the following model was established:

COX_RNTIC_ = 0.053 × **R**DW + 0.027 × **N**LR + 0.003 × **T**BIL+ 0.317 ×**I**NR + 0.003 × **C**r with a cut-off value of 3.08 (sensitivity: 77.89%, specificity: 86.04%). The model was able to predict 190 patients alive and 155 dead, accurately classifying 81.95% of the patients in this study (Table 3).

**Table 2:** Cox regression analysis for variables associated with 90-day mortality in patients with HBV-ACLF

Variables	Univariate analysis		Multivariate analysis		
	HR 95%(CI)	*P* value	Beta coefficient	HR 95%(CI)	*P* value
TBIL (μmol/L)	1.005 (1.004–1.005)	< 0.001	0.003	1.002 (1.001–1.003)	< 0.001
Cr (μmol/L)	1.008 (1.005–1.010)	< 0.001	0.003	1.003 (1.001–1.005)	0.001
Cyst-c (mg/L)	1.055 (1.014–1.097)	0.008			
Serum Na^+^(mmol/L)	0.981 (0.959–1.004)	0.112			
INR	1.488 (1.342–1.651)	< 0.001	0.317	1.318 (1.163–1.494)	< 0.001
PTA (%)	0.973 (0.959–0.988)	< 0.001			
WBC (10^9^/L)	1.068 (1.043–1.094)	< 0.001			
Neutrophils (10^9^/L)	1.082 (1.055–1.110)	< 0.001			
Monocytes(10^9^/L)	1.080 (0.997–1.170)	0.060			
Lymphocytes(10^9^/L)	0.856 (0.640–1.144)	0.293			
RDW (%)	1.112 (1.075–1.15)	< 0.001	0.053	1.047 (1.009–1.086)	0.015
NLR	1.053 (1.038–1.068)	< 0.001	0.027	1.027 (1.009–1.046)	0.003
MLR	1.057 (1.007–1.109)	0.025			
PLR	1.003 (1.001–1.005)	0.001			
RLR	1.016 (1.007–1.026)	0.001			
HE	1.221				
	(1.101–1.353)	< 0.001			
Bacterial infection	0.55				
	(0.383–0.785)	0.001			

*TBIL* total bilirubin, *ALB* albumin, *Cr* creatinine, *Cyst-c* Cystatin c, *Serum Na*^*+*^ serum sodium, *PT* prothrombin time, *INR* international normalized ratio, *PTA* prothrombin activity, *WBC* white blood cell count, *RDW* red blood cell distribution width, *NLR* neutrophil/lymphocyte ratio, *MLR* monocyte/lymphocyte ratio, *PLR* platelet/lymphocyte ratio, *RLR* RDW/lymphocyte ratio, *HE* Hepatic encephalopathy, *HBV-ACLF* hepatitis B virus related acute-on-chronic liver failure

**Table 3:** Comparison of predictive value of RNTIC, MELD, MELD-Na and CTP in derivation cohort and validation cohort

Variables	AUC (95%)	Z Statistic	*P* Value	Cut-off Value	Sensitivity (%)	Specificity (%)	PPV (%)	NPV (%)	Overall Accuracy (%)	Youden Index
Derivation cohort										
RNTIC	0.873 (0.837–0.903)			3.08	77.89	86.04	82.89	81.2	81.95	0.64
MELD	0.732 (0.687–0.774)	8.227	<0.001	24.14	70.35	70.72	68.29	72.69	70.54	0.41
MELD-Na	0.714 (0.668–0.757)	6.868	<0.001	18.00	84.4	50.50	60.21	78.17	66.27	0.35
CTP	0.703 (0.657–0.747)	6.424	<0.001	10.00	82.91	47.30	58.51	75.54	64.13	0.30
Validation cohort										
RNTIC	0.845 (0.778–0.898)			2.59	85.07	71.00	76.25	80.26	78.21	0.62
MELD	0.768 (0.694–0.832)	2.867	0.004	22.24	69.64	83.00	68.42	82.83	77.56	0.53
MELD-Na	0.759 (0.684–0.824)	2.952	0.003	19.12	73.21	71.00	70.83	70.24	70.51	0.44
CTP	0.718 (0.641–0.787)	2.903	0.003	10.00	75.00	63.00	65.82	68.83	67.31	0.38

Z Statistic: compared with AUC of RNTIC, P value: compared with AUC of RNTIC, RNTIC = 0.053 × RDW + 0.027 × NLR + 0.003 × TBIL+ 0.317 × INR + 0.003 × Cr, *MELD* model for end-stage liver disease, *CTP* child-Turcotte Pugh score, *MELD-Na* MELD-sodium score, *NLR* neutrophil/lymphocyte ratio, *RDW* red blood cell distribution width, *TBIL* total bilirubin, *Cr* creatinine, *INR* international normalized ratio, *HBV-ACLF* hepatitis B virus related acute-on-chronic liver failure, *NPV* negative predictive value, *PPV* positive predict value

### Comparison of predictive value of MELD score, MELD-Na, CTP and RNTIC for prognosis of patients with HBV-ACLF

**Fig. 3:**
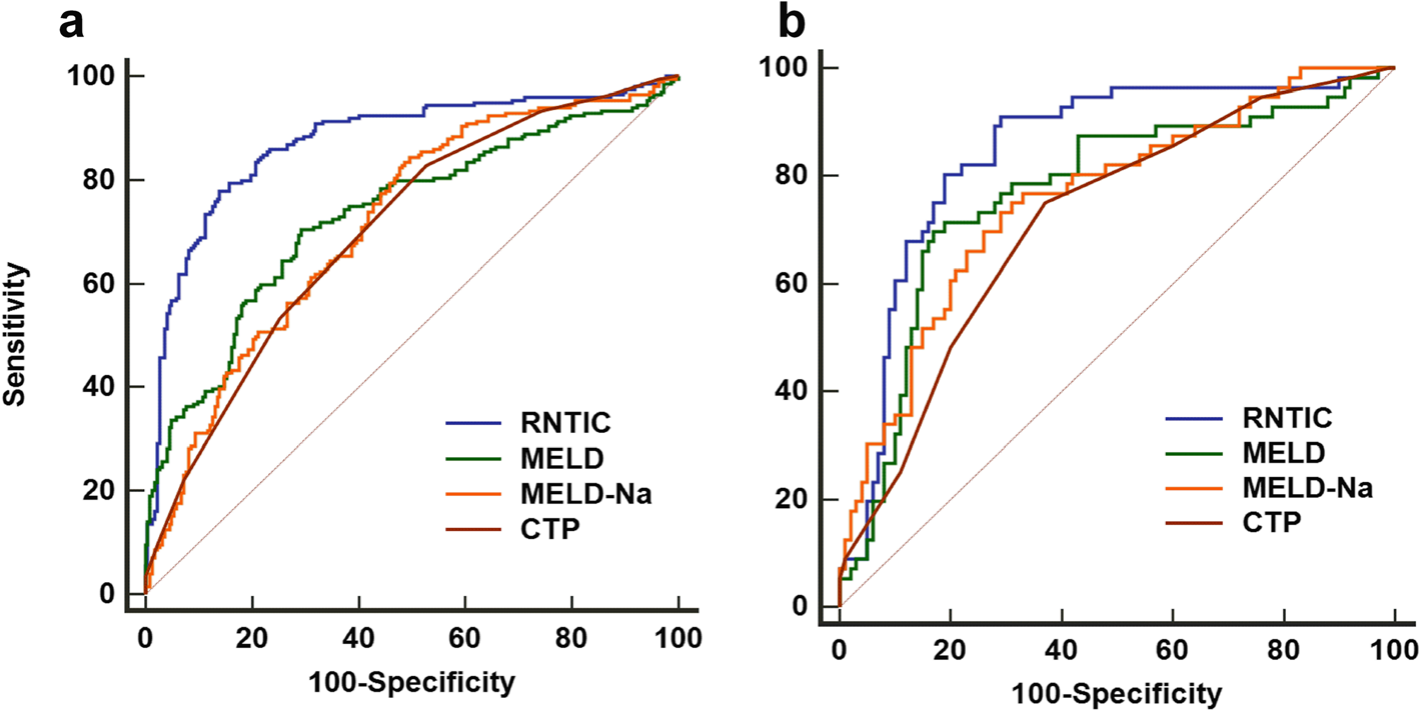
(**a**) Receiver operating characteristics (ROC) curve analysis for prediction of 90-day mortality by RNTIC, MELD, MELD-Na and CTP in derivation cohort; (**b**) Receiver operating characteristics (ROC) curve analysis for prediction of 90-day mortality by RNTIC, MELD, MELD-Na and CTP in validation cohort

Receiver operating characteristic (ROC) curves for parameters including MELD scores, MELD-Na, CTP and RNTIC were shown in Fig. 3a. RNTIC had a higher area under the ROC curve (AUC) for identifying poor prognosis than the other three (*p* < 0.001, Table 3).

### External validation of RNTIC

In order to test the model, 180 patients were enrolled from the other two hospitals. According to the inclusion and exclusion criteria, 156 patients were admitted to the validation cohort (Fig. 1) with a 90-day mortality rate at 35.89%. Comparisons of demographics and baseline clinical characteristics of the patients in the derivation and validation cohort were summarized in Table 4. The AUC of the RNTIC was higher than MELD, MELD-Na and CTP (*P* < 0.05, Fig. 3b, Table 3), which proved this model also had an efficient ability on the prediction of the 90-day death in patients with HBV-ACLF in the validation cohort.

**Table 4:** Comparisons of demographics and baseline clinical characteristics of the patients in the derivation and validation cohort

Variables	Validation cohort(*n* = 156)	derivation cohort (*n* = 421)	*P* value
Age (years)	48.92 ± 11.94	47.93 ± 11.40	0.372
Gender (M: F)	129/27	365/56	–
Cirrhosis (%)	114(73.1%)	299(71.02%)	0.627
Bacterial infections	101(64.74%)	307(72.92%)	0.055
TBIL (μmol/L)	239.43 ± 136.17	323.39 ± 165.69	< 0.001
INR	1.92(1.58, 2.43)	2.25(1.86, 2.76)	< 0.001
Cr (μmol/L)	62.40(52.15, 80.35)	69.30(57.28, 87.90)	0.004
RDW (%)	16.10(14.50, 18.10)	15.90(14.38, 18.42)	0.842
MPV (FL)	11.29 ± 1.67	11.74 ± 1.53	0.003
NLR	4.33(2.68, 7.87)	4.84(3.19, 8.07)	< 0.001
MLR	0.61(0.41, 0.90)	0.64(0.42, 0.94)	0.473
GPR	0.91(0.53, 1.50)	0.97(0.59, 1.69)	0.162
RPR	0.17(0.11, 0.28)	0.19(0.12, 0.30)	0.093
MPR	0.70(0.59, 0.83)	0.14(0.09, 0.20)	< 0.001
PNI	35.03(30.65, 41.10)	34.10(30.20, 38.31)	0.033
PLR	95.92(63.99, 143.22)	91.03(62.69, 127.69)	0.206
RLR	17.77(11.73, 26.73)	16.70(3.71, 85.81)	0.939
MELD SCORE	21.41 ± 7.84	25.05 ± 6.75	< 0.001
MELD-Na	21.24 ± 15.66	24.32 ± 12.69	0.001
CTP	10.48 ± 1.89	11.09 ± 2.00	0.029

*TBIL* total bilirubin, *ALB* albumin, *γ-GGT* gamma-glutamyl transpeptidase, *Cr* creatinine, *Cyst-c* Cystatin c, *Serum Na +* serum sodium, *PT* prothrombin time, *INR* international normalized ratio, *PTA* prothrombin activity, *WBC* white blood cell count, *RDW* red blood cell distribution width, *NLR* neutrophil/lymphocyte ratio, *MLR* monocyte/lymphocyte ratio, *PLR* platelet/lymphocyte ratio, *RPR* RDW/platelet ratio, *GPR* gamma-glutamyl transpeptidase/platelet ratio, *MPV* mean platelet volume, *RLR* RDW/lymphocyte ratio, *PNI* prognostic nutritional index, *MPR* MPV/platelet ratio, *MELD* model for end-stage liver disease, *MELD-Na* MELD-sodium score, *CTP* child-Turcotte Pugh score

## Discussion

ACLF with a high mortality is a systemic inflammatory response driven by cytokines secretion, oxidative stress, immune dysfunction and increased risk of infection, which also compromises organ function integrity [21, 22]. In this study, a triple-center retrospective research was launched to create a new prognostic model taking inflammatory markers into consideration for patients with HBV-ACLF. Compared the routine hematological inflammatory parameters listed in Table 1, We found only NLR and RDW were independent prognostic factors associated with 90-day mortality in patients with HBV-ACLF, and then combined RDW and NLR with other three statistically significant indicators (TBIL, INR, Cr) to establish a new prognostic model, which performed a better predictive value both in derivation and validation cohort. In addition, the study also analyzed the 28-day prediction ability of the inflammatory marker-based model. Compared with the prediction of 90-day viability, all models showed a poor ability to predict 28-day patient survival. Additional files showed this in more detail (see Additional files 1 and 2).

It was reported that increased neutrophil counts reflected oxidative stress and that lower lymphocyte counts reflected a deterioration of nutritional status [23]. Thus, the Neutrophil and lymphocyte counts could reflect inflammation status and general nutrition status of patients. The NLR has been researched in many diseases including liver disease. Increased NLR is predictive of mortality in advanced illnesses apart from infections including malignancy, acute coronary syndrome, intracerebral hemorrhage, chronic kidney disease and rheumatic diseases [24, 25], and elevated NLR has a tight relationship with the prognosis of hepatitis, liver cirrhosis and liver cancer [14, 26, 27]. In our study, the NLR value significantly elevated in the HBV-ACLF death group, and was an independent risk factor for 90-day death in HBV-ACLF patients, which was consistent with the study by Cai J, et al. [15], but the specific mechanism of HBV-ACLF patients’ poor prognosis and NLR elevation is unclear. It was reported that in patients with end-stage liver disease, the body’s immune system and inflammatory response were over-activated with a large number of inflammatory factors being released into the bloodstream (e.g., IL-6, IL-8, TNF-α, etc.) [28], which caused damage to hepatocytes. Moreover, the robust inflammatory reaction could cause amounts of lymphocyte apoptosis, and make neutrophils originally presented in the hepatic sinusoids released into the blood, thereby increasing the level of NLR [29].Thus, the hypothesis that elevated NLR reflects the severity of the potentially acute systemic inflammation following primary injury is widely accepted.

In addition, another inflammatory marker RDW also was proved to be an independent risk factor for 90-day death in HBV-ACLF patients. However, the reason why RDW elevation is closely associated with the outcome of the patients with HBV-ACLF is still unclear. It may be due to the following five reasons: (I) Significant changes in RDW are associated with some abnormalities, such as inflammation, oxidative stress, red blood cell fragmentation, poor nutritional status, and erythropoietin dysfunction [30]. Pro-inflammatory factors could damage the maturation of red blood cells and cause immature red blood cells to enter the bloodstream simultaneously, leading to an increase in RDW [8]. (II) Inflammatory cytokines such as tumor necrosis factor TNF-α, IL-1β and IL-6 may inhibit iron metabolism and erythropoietin production, leading to synthetic disorders or abnormal erythropoietin activity [31]. (III) Excessive hepatocyte necrosis resulting in decreased liver reservation of vitamin B12, folic acid and iron [32], elevated the RDW. (IV) Pathological immune response to HBV can release inflammatory mediators and endotoxin etc., which affect the growth and development of red blood cells, making RDW rise [8]. (V) Low serum antioxidant concentrations characterized by a compromise between oxidant and antioxidant defenses are associated with increased levels of RDW, which is common in liver disease [33].

Apart from the inflammatory markers NLR and RDW, we also found that Cr, TBIL, and INR, reflecting the function of liver, kidney, and coagulation in routine hematological tests, were independent risk factors for prognosis of HBV-ACLF in this study. Therefore, the new prediction Cox regression model was constructed based on the above five indicators, which showed a great predictive performance both in the derivation and validation cohort with high sensitivity and specificity.

Some limitations of our study must be considered. First, this was a retrospective study, so we did not observe the changes of RDW and NLR values dynamically. In the future, more prospective studies are needed to reveal the association between RDW, NLR longitudinal changes and outcomes in HBV-ACLF patients. Second, we did not test other pro-inflammatory cytokines, such as TNF-α, IL-1β, IL-6, and IL-8, which may contribute to revealing the mechanisms. Third, the study only analyzed the patients hospitalized in ward and had not analyzed the patients on dialysis under mechanical ventilation. Forth, due to the retrospective study design, some data lacking led that kinds of scoring systems for ACLF such as APASL ACLF Research Consortium (AARC), the Chinese Group on the Study of Severe Hepatitis B (COSSH) and ClIF-SOFA cannot be analyzed.

## Conclusions

In summary, compared with the MELD score, MELD-Na and CTP, our newly established model has a better predictive ability to assess the 90-day mortality in HBV-ACLF patients in the early stage.

## Supplementary information

**Additional file 1: Additional Table 1**. The Comparison of 28-day with 90-day predictive value of RNTIC, MELD, MELD-Na and CTP in derivation cohort. Z Statistic: compared with AUC of RNTIC, *P* value: compared with AUC of RNTIC, RNTIC = 0.053 × RDW + 0.027 × NLR + 0.003 × TBIL+ 0.317 × INR + 0.003 × Cr, MELD = model for end-stage liver disease, CTP = child-Turcotte Pugh score, MELD-Na = MELD-sodium score, NLR = neutrophil/lymphocyte ratio, RDW = red blood cell distribution width, TBIL = total bilirubin, Cr = creatinine, INR = international normalized ratio, HBV-ACLF = hepatitis B virus related acute-on-chronic liver failure, NPV = negative predictive value, PPV = positive predict value.

**Additional file 2: Additional Figure 1.** Receiver operating characteristics (ROC) curve analysis for prediction of 28-day mortality by RNTIC, MELD, MELD-Na and CTP in derivation cohort.

## Data Availability

The datasets analyzed during the current study are not publicly available because they contain sensitive patient information, but may available from the corresponding author on reasonable request.

## Abbreviations

NLR: neutrophil/lymphocyte ratio
RDW: red blood cell distribution width
HBV-ACLF: hepatitis B virus-related acute-on-chronic liver failure
AUC: the area under the receiver operating characteristics curve
CLIF-SOFA: chronic liver failure sequential organ failure assessment
CTP: child-Turcotte Pugh score
MELD: model for end-stage liver disease score
MELD-Na: MELD-sodium score
HBV: hepatitis B virus
MPV: mean platelet volume
MLR: monocyte/lymphocyte ratio
PLR: platelet/lymphocyte ratio
RPR: RDW/platelet ratio
GPR: gamma-glutamyl transpeptidase/platelet ratio
RLR: RDW/lymphocyte ratio
PNI: prognostic nutritional index
MPR: MPV/platelet ratio
IL-6: Interleukin-6
TBIL: total bilirubin
INR: international normalized ratio of prothrombin time
PTA: prothrombin activity
ROC: receiver operating characteristic analysis
ALT: alanine aminotransferase
AST: aspartate aminotransferase
ALB: albumin
γ-GGT: gamma-glutamyl transpeptidase
Cr: creatinine
Cyst-c: cystatin c
Serum k^+^: serum potassium
Serum Na^+^: serum sodium
PCT: procalcitonin
HE: Hepatic encephalopathy
NPV: negative predictive value
PPV: positive predict value
AARC: APASL ACLF Research Consortium
COSSH: the Chinese Group on the Study of Severe Hepatitis B
