# Supplementary material for: A novel predictive model based on inflammatory markers to assess the prognosis of patients with HBV-related acute-on-chronic liver failure: a retrospective cohort study
Source: BMC Gastroenterol. 2020 Sep 16;20:301. doi: 10.1186/s12876-020-01437-2 (PMC7493843; doi:10.1186/s12876-020-01437-2)
Supplement: Supplementary file 1 — Additional file 1: Additional Table 1. The Comparison of 28-day with 90-day predictive value of RNTIC, MELD, MELD-Na and CTP in derivation cohort. Z Statistic: compared with AUC of RNTIC, P value: compared with AUC of RNTIC, RNTIC = 0.053 × RDW + 0.027 × NLR + 0.003 × TBIL+ 0.317 × INR + 0.003 × Cr, MELD = model for end-stage liver disease, CTP = child-Turcotte Pugh score, MELD-Na = MELD-sodium score, NLR = neutrophil/lymphocyte ratio, RDW = red blood cell distribution width, TBIL = total bilirubin, Cr = creatinine, INR = international normalized ratio, HBV-ACLF = hepatitis B virus related acute-on-chronic liver failure, NPV = negative predictive value, PPV = positive predict value. [file 12876_2020_1437_MOESM1_ESM.docx]

| Variables | AUC  (95%) | Z Statistic | P Value | Cut-off Value | Sensitivity  (%) | Specificity  (%) | PPV  (%) | NPV  (%) | Overall  Accuracy  (%) | Youden  Index |
| --- | --- | --- | --- | --- | --- | --- | --- | --- | --- | --- |
| 90-dayt | | | | | | | | | | |
| RNTIC | 0.873  (0.837-0.903) |  |  | 3.08 | 77.89 | 86.04 | 82.89 | 81.2 | 81.95 | 0.64 |
| MELD | 0.732  (0.687-0.774) | 8.227 | ＜0.001 | 24.14 | 70.35 | 70.72 | 68.29 | 72.69 | 70.54 | 0.41 |
| MELD-Na | 0.714  (0.668-0.757) | 6.868 | ＜0.001 | 18.00 | 84.4 | 50.50 | 60.21 | 78.17 | 66.27 | 0.35 |
| CTP | 0.703  (0.657-0.747) | 6.424 | ＜0.001 | 10.00 | 82.91 | 47.30 | 58.51 | 75.54 | 64.13 | 0.30 |
| 28-day | | | | | | | | | | |
| RNTIC | 0.727  (0.682-0.769 |  |  | 3.76 | 52.73 | 86.61 | 37.18 | 92.42 | 82.18 | 0.39 |
| MELD | 0.663  (0.616-0.708) | 2.785 | 0.005 | 28.63 | 52.73 | 80.05 | 28.43 | 91.85 | 76.48 | 0.33 |
| MELD-Na | 0.638  (0.590-0.684) | 2.912 | 0.003 | 30.34 | 47.27 | 75.68 | 22.60 | 88.56 | 70.54 | 0.23 |
| CTP | 0.594  (0.546-0.642) | 2.779 | 0.006 | 12.00 | 29.09 | 87.98 | 26.67 | 89.20 | 80.28 | 0.17 |
